# Organization and regulation of the actin cytoskeleton in the pollen tube

**DOI:** 10.3389/fpls.2014.00786

**Published:** 2015-01-08

**Authors:** Xiaolu Qu, Yuxiang Jiang, Ming Chang, Xiaonan Liu, Ruihui Zhang, Shanjin Huang

**Affiliations:** ^1^Center for Plant Biology, School of Life Sciences, Tsinghua UniversityBeijing, China; ^2^Key Laboratory of Plant Molecular Physiology, Institute of Botany – Chinese Academy of SciencesBeijing, China

**Keywords:** actin, pollen tube, actin-binding protein, formin, villin, ADF, fimbrin

## Abstract

Proper organization of the actin cytoskeleton is crucial for pollen tube growth. However, the precise mechanisms by which the actin cytoskeleton regulates pollen tube growth remain to be further elucidated. The functions of the actin cytoskeleton are dictated by its spatial organization and dynamics. However, early observations of the distribution of actin filaments at the pollen tube apex were quite perplexing, resulting in decades of controversial debate. Fortunately, due to improvements in fixation regimens for staining actin filaments in fixed pollen tubes, as well as the adoption of appropriate markers for visualizing actin filaments in living pollen tubes, this issue has been resolved and has given rise to the consensus view of the spatial distribution of actin filaments throughout the entire pollen tube. Importantly, recent descriptions of the dynamics of individual actin filaments in the apical region have expanded our understanding of the function of actin in regulation of pollen tube growth. Furthermore, careful documentation of the function and mode of action of several actin-binding proteins expressed in pollen have provided novel insights into the regulation of actin spatial distribution and dynamics. In the current review, we summarize our understanding of the organization, dynamics, and regulation of the actin cytoskeleton in the pollen tube.

## INTRODUCTION

Pollen represents a critical stage of the plant life cycle and is essential for the production of seeds in flowering plants (McCormick, 2013). Upon landing on the surface of the stigma, pollen begins to hydrate and germinate, protruding outgrowth to form a tubular structure that extends rapidly in the style. This structure provides the passage for two non-motile sperm cells to be delivered to the female gametophyte and finally effect the double fertilization (Hepler et al., 2001; Lord and Russell, 2002; Cheung and Wu, 2008). Pollen tube growth is very rapid; the growth rate for maize pollen tubes in the style can reach up to 1 cm/h (Bedinger, 1992). During the journey of fertilization, the pollen tube normally traverses a distance 1000s of times the diameter of its grain. However, growth of the pollen tube is restricted to the tip region, which is therefore called “tip growth”. This type of tip growth found in the pollen tube is shared by several other cell types, including root hairs in plants, protonemal cells in moss, hyphae in fungi, and neurites in animals (Cheung and Wu, 2008; Rounds and Bezanilla, 2013). Among these systems, pollen tube growth is particularly analogous to neurite growth. Surprisingly, however, despite its rigid cell wall, the pollen tube extends very fast, e.g., the lily pollen tubes even grow one order of magnitude faster than neurite (Hepler et al., 2001). The rapidity of growth implies that the underlying cellular activities may be amplified in the pollen tube. Pollen is an excellent cellular model for study of tip growth, as it is easy to culture, and most of the features associated with *in vivo* growth of pollen tubes are also observed *in vitro*. Additionally, essential mutations associated with pollen function can be maintained under a heterozygous state, which makes pollen tube a very nice genetic system to study polarized cell growth. For these reasons, over the past several decades, the pollen tube has served a very important model cellular system for intensive study of the mechanisms underlying polarized cell growth.

The actin cytoskeleton has been shown to be crucial for pollen tube growth (Taylor and Hepler, 1997; Gibbon et al., 1999; Hepler et al., 2001; Vidali et al., 2001; Smith and Oppenheimer, 2005; Hussey et al., 2006; Chen et al., 2009; Fu, 2010; Staiger et al., 2010). The precise molecular mechanisms underlying the function of the actin cytoskeleton in the pollen tube, however, remain poorly understood. Different models have been proposed regarding the function of the actin cytoskeleton during pollen tube growth. One of the more common ideas is that the actin cytoskeleton drives the intracellular transport system that carries Golgi-derived vesicles containing the materials necessary for cell wall synthesis and membrane fusion to the tip (Pierson and Cresti, 1992; Hepler et al., 2001; Vidali and Hepler, 2001). The actin cytoskeleton has also been viewed as a structural element that supports the turgor pressure needed to drive and maintain rapid pollen tube growth (Picton and Steer, 1982; Steer and Steer, 1989; Derksen et al., 1995). Additionally, actin polymerization itself has also been shown to be important for pollen tube growth (Gibbon et al., 1999; Vidali et al., 2001). Irrespective of the particular mechanism underlying the function of the actin cytoskeleton in polarized growth, it is important to precisely describe its spatial distribution and dynamics in the pollen tube. Since, the spatial distribution and dynamics of the actin cytoskeleton are modified by the presence of various actin-binding proteins (ABPs) in cells (Staiger et al., 2010; Huang et al., 2014), it is also important to characterize the function and mode of action of these ABPs. Indeed, recent characterization of the mode of action of several pollen-expressed ABPs has provided exceptional insights into the regulation of actin organization and dynamics in the pollen tube. Thus, the purpose of this review is to summarize our current understanding of the organization and dynamics of the actin cytoskeleton, as well as its regulation, in the pollen tube.

## SPATIAL DISTRIBUTION OF ACTIN FILAMENTS IN THE POLLEN TUBE

The distributions of the actin cytoskeleton in fixed pollen tubes from different species have been characterized using immunostaining with anti-actin antibodies and staining with fluorescent phalloidin (Tang et al., 1989; Rutten and Derksen, 1990; Gibbon et al., 1999; Geitmann et al., 2000; Li et al., 2001; Ye et al., 2009). Historically, reaching a consensus with respect to the distribution of actin filaments in the apical and subapical regions has been quite problematic. Different results have been reported regarding the distribution of actin filaments in the apical and subapical regions (Derksen et al., 1995; Miller et al., 1996). The variation could be due to differences between species or due to the use of different staining methods. The variation in actin structures in the apical region of pollen tubes stained using different methods most likely results from alterations in the fixation step, in which apical actin filaments may not be well-preserved. This problem is presumably due to two factors: one is that the pollen tube grows too rapidly and cannot be fixed instantaneously, and another is that apical actin filaments are highly dynamic and fragile. The original observations from experiments using conventional fixation procedures showed that dense actin filaments are present in the tip (Tiwari and Polito, 1988; Tang et al., 1989; Derksen et al., 1995). However, injection of rhodamine phalloidin into pollen tubes failed to label actin filaments in the extreme apex (Miller et al., 1996), and several other studies showed that the extreme apex is depleted of actin filaments in fixed pollen tubes (Li et al., 2001; Raudaskoski et al., 2001; Vidali et al., 2001). Later, using improved fixation regimens more likely to better preserve the cellular structures, such as rapid freeze fixation, the distribution of the actin cytoskeleton in the apical region was reproducibly revealed in the pollen tube (Lovy-Wheeler et al., 2005). This actin distribution pattern was further confirmed by the results of actin filament labeling using live-cell actin markers (Kost et al., 1998; Fu et al., 2001; Qu et al., 2013; see also the description below).

**FIGURE 1:**
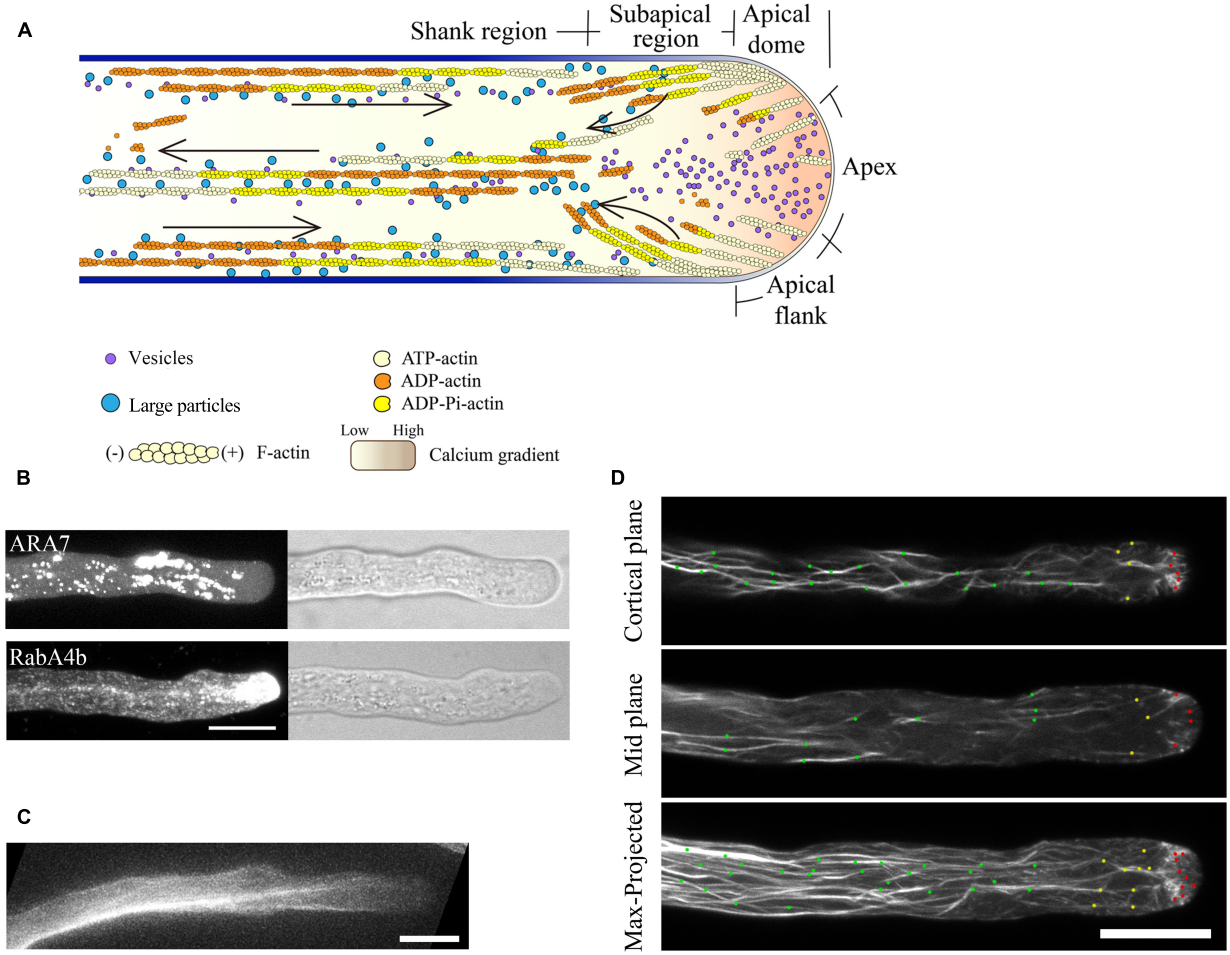
**Spatial distribution of actin filaments in the pollen tube. (A)** Schematic showing the spatial distribution of actin filaments in the pollen tube. At the apex, actin filaments are less abundant. In the subapical region, actin filaments form the regular actin collar structure. In the shank region, actin filaments are axially packed into cables, termed longitudinal actin cables. These actin structures are believed to perform distinct functions. Longitudinal actin cables provide the molecular tracks for movement of large organelles and vesicles from the base to the tip. The organelles and vesicles reverse direction at the subapical region and return to the base via the middle of the pollen tube, giving rise to the reverse-fountain cytoplasmic streaming pattern. These large organelles never enter the tip, resulting in the formation of the optical smooth zone at the tip referred to as the “clear zone”. However, this region is filled with small vesicles. Therefore, actin filaments at the apical region are believed to be important for vesicle targeting and fusion events. The black arrows indicate the direction of cytoplasmic streaming. **(B)** Spatial distribution of ARA7-positive vesicles and RabA4b-positive vesicles in pollen tubes. ARA7-positive vesicles do not invade the apical region, whereas RabA4b-positive vesicles enter the apical region. Scale bar = 10 μm. **(C)** ABD2-GFP decorates longitudinal actin cables. Scale bar = 10 μm. **(D)** Actin filaments in *Arabidopsis* pollen tubes were revealed by decoration with Lifeact-mEGFP. Images showing the cortical plane, the middle plane, and a projection of a representative pollen tube are presented. Actin filaments in the shank region, subapical region, and apical region are indicated by green dots, yellow dots, and red dots, respectively. Scale bar = 10 μm.

Thus, the current consensus view of the spatial distribution of actin filaments is that they are arrayed into at least three distinct structures in the pollen tube, consistent with the zonation of cytoplasm (**Figure 1A**; Lovy-Wheeler et al., 2005; Ren and Xiang, 2007; Chen et al., 2009; Staiger et al., 2010). In the shank, actin filaments are arranged axially into bundles with uniform polarity, which allows the transport of organelles or vesicles from the base to the tip along the cell cortex. At the subapex, actin filaments form regular structures referred to as the collar (Gibbon et al., 1999; Fu et al., 2001), fringe (Lovy-Wheeler et al., 2005), mesh (Geitmann et al., 2000; Chen et al., 2002), or funnel (Vidali et al., 2001) in pollen tubes from different species. In this region, cytoplasmic streaming reverses direction and turns back toward the base along the axial actin cables in the center of the tube, giving rise to the reverse-fountain cytoplasmic streaming pattern (Hepler et al., 2001; Ye et al., 2009). Though large organelles do not enter the apical region, small vesicles enter into and become accumulated in the apical region (**Figures 1A,B**). In the apical region, actin filaments are less abundant, but are highly dynamic. The dynamics of the tip-localized population of actin filaments have been investigated in tobacco, lily, and *Arabidopsis* pollen tubes (Fu et al., 2001; Qu et al., 2013; Rounds et al., 2014 see also the detailed description below), which has expanded our understanding of the function of actin filaments. However, the means by which those actin filaments precisely regulate underlying cellular events, like vesicle targeting and fusion, remains to be explored.

## ACTIN MARKERS USED TO DECORATE ACTIN FILAMENTS IN GROWING POLLEN TUBES

Staining of actin filaments in fixed pollen tubes has yielded a great deal of useful information regarding the spatial distribution of actin filaments in the pollen tube (Gibbon et al., 1999; Geitmann et al., 2000; Lovy-Wheeler et al., 2005). However, the use of fixed tissues provides only a static image and does not reveal how the actin cytoskeleton is remodeled during pollen tube growth. Therefore, development of markers to label actin filaments in living pollen tubes was much needed, in order to allow tracing of actin filament dynamics during pollen tube growth. The introduction of green fluorescent protein (GFP) has revolutionized the way in which cellular dynamics are visualized (Brandizzi et al., 2002). Numerous useful GFP fusion markers have been developed in order to visualize cytoskeletal dynamics and have revolutionized research in the cytoskeleton field (Stringham et al., 2012). In plants, to date, GFP-actin has never been demonstrated to be a useful marker for decorating filamentous actin, presumably due to one or more of the following reasons. One possible issue is that the GFP tag alters the function of actin and prevents its incorporation into filamentous actin or alters the conformation of filamentous actin after its incorporation. Another possible reason is that filamentous actin only represents a small population of the total actin in plant cells (Staiger and Blanchoin, 2006); therefore, the filamentous signal might be masked by the overwhelming amount of monomeric signal.

Tagging of some ABPs or the actin-binding domains derived from them with GFP has provided a non-invasive way to image actin filaments in plants. Several of these actin markers have been used to image actin filaments in growing pollen tubes (**Table 1**). The earliest actin marker used for decorating actin filaments in pollen tubes was GFP-mTalin (**Table 1**; Kost et al., 1998). However, nowadays, GFP-mTalin is rarely used because it causes excessive filament bundling (Ketelaar et al., 2004b). GFP-ADF was shown to decorate actin filaments and most prominently at the subapical region of the pollen tube (**Table 1**; Chen et al., 2002; Cheung et al., 2008, 2010), and LIM-GFP and GFP-fimbrin/ABD2-GFP decorate longitudinal actin cables in the shanks of pollen tubes (**Table 1**; **Figure 1C**; Wilsen et al., 2006; Cheung et al., 2008). However, these actin markers each have distinct disadvantages in revealing actin structures in the pollen tube; for example, GFP-fimbrin/ABD2-GFP does not label actin filaments well in the apical and subapical regions (**Figure 1C**; Cheung et al., 2008). Despite these issues, these markers are useful for labeling different aspects of the actin cytoskeleton. Thus, the use of different actin markers can be effective for study of the distribution and changes in the actin cytoskeleton in the pollen tube. However, the ideal actin marker would be able to detect all arrays of actin filaments present in the growing pollen tube, and it would be even better if it had minimal effect on normal actin dynamics. In this regard, Lifeact-mEGFP has become the actin marker of choice in the pollen tube. Lifeact-mEGFP contains an actin-binding site consisting of 17 amino acids derived from yeast ABP-140 fused with mEGFP. This protein decorates actin filaments in animal cells (Riedl et al., 2008) and has been used to detect actin filaments in growing tobacco and lily pollen tubes (Vidali et al., 2009; Dong et al., 2012). Recently, it has been employed to detect actin filaments in *Arabidopsis* pollen tubes (Qu et al., 2013; Zhu et al., 2013; Qin et al., 2014). Lifeact-mEGFP reveals actin structures nicely within different regions of the *Arabidopsis* pollen tube (**Figure 1D**; Qu et al., 2013), and results using this marker are reminiscent of results from actin staining of fixed pollen tubes (Gibbon et al., 1999; Lovy-Wheeler et al., 2005). Therefore, Lifeact-mEGFP represents an ideal actin marker for visualization of the organization and tracing of the dynamics of actin filaments in the pollen tube (**Figure 1D**; see the following section). Certainly, careful analysis of the organization and dynamics of the actin cytoskeleton in the entire pollen tube using a combination of standard and new actin markers will be useful in the future.

**Table 1:** Actin markers used to decorate actin filaments in living pollen tubes.

Actin markers	Pollen tubes	Reference
GFP-mTalin	Tobacco and *Arabidopsis*	Kost et al. (1998); Fu et al. (2001), Ketelaar et al. (2004b); Wilsen et al. (2006), Wang et al. (2008a); Zhang et al. (2009, 2010b), Gui et al. (2014)
GFP-FIMBRIN/ABD2-GFP	Tobacco and lily	Wilsen et al. (2006); Liao et al. (2010)
GFP-ADF	Tobacco and lily	Chen et al. (2002); Cheung and Wu (2004), Wilsen et al. (2006); Cheung et al. (2008, 2010)
LIM-GFP	Tobacco	Cheung et al. (2008)
Lifeact-mEGFP	Tobacco, lily, and *Arabidopsis*	Vidali et al. (2009); Daher and Geitmann (2011), Dong et al. (2012); Qu et al. (2013), Zhu et al. (2013); Gui et al. (2014), Qin et al. (2014); Rounds et al. (2014)

## ACTIN FILAMENT DYNAMICS IN THE POLLEN TUBE

The actin cytoskeleton plays an integral role during pollen tube growth. It is well-appreciated that the relatively stable longitudinal actin cables drive intracellular movement in the shank to propel pollen tube growth (Hepler et al., 2001; Ye et al., 2009; Wu et al., 2010). Meanwhile, several studies have indicated that the dynamic state of actin filaments in the tip region is also crucial for growth (Gibbon et al., 1999; Vidali et al., 2001). Apical actin filaments are thought to play an important role in regulating the velocity and direction of pollen tube growth by controlling vesicle docking and fusion events (Gibbon et al., 1999; Fu et al., 2001; Vidali et al., 2001; Lee et al., 2008; Qu et al., 2013). However, the precise functioning of actin filaments within the apical region remains poorly understood. This is partly because we know very little about the precise organization of the apical actin filaments. Direct visualization of individual actin filaments and quantification of the associated parameters are needed to provide insights into the organizational nature of these apical actin filaments.

**FIGURE 2:**
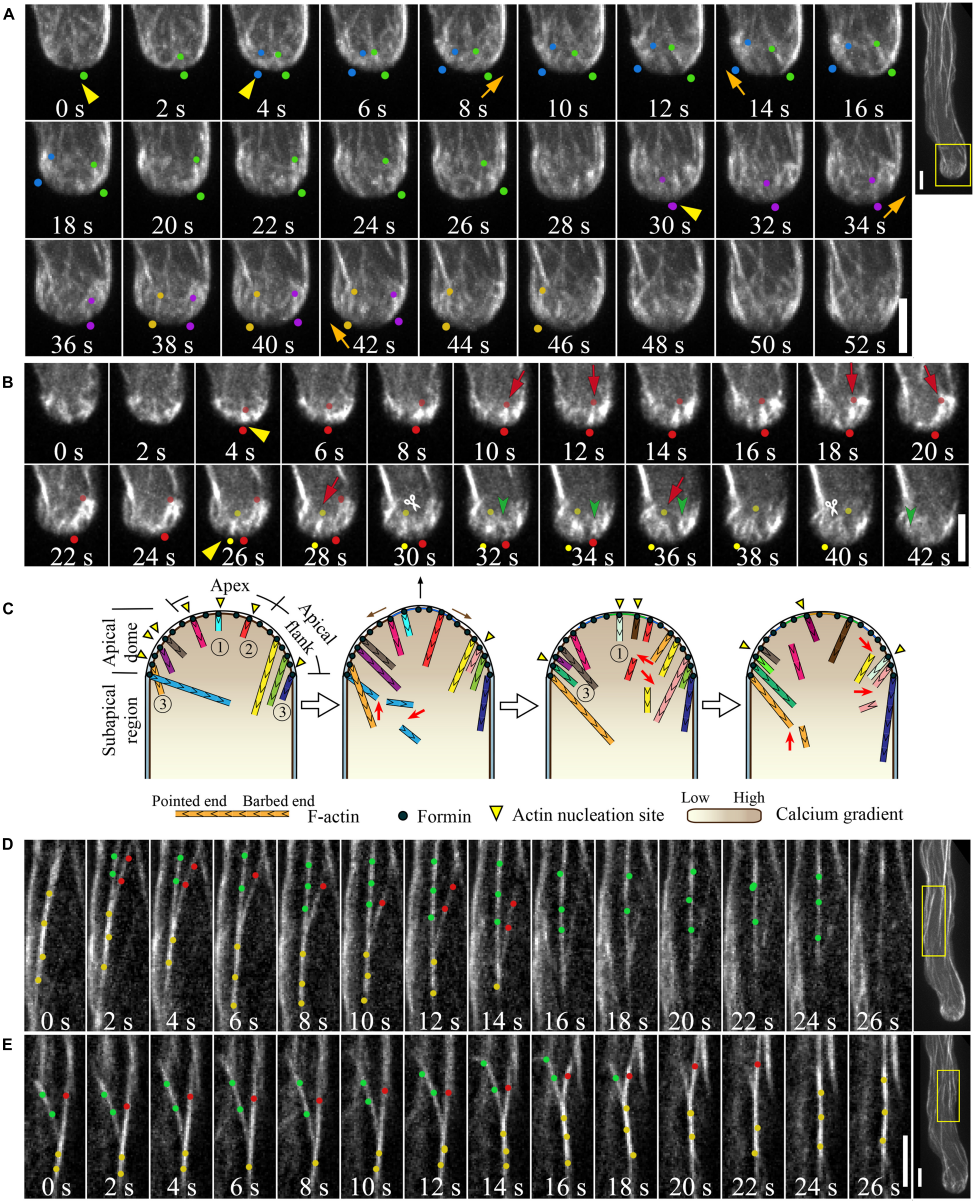
**Actin dynamics in the pollen tube. (A)** Actin filaments are constantly generated from the apical membrane within the apical dome. The images presented are maximally projected time-lapse images. Emerging individual actin filaments are marked by two dots of the same color. Yellow triangles indicate the origination of actin polymerization events, and movement of the filaments from the apex to the apical flank is indicated by orange arrows. Images are a higher magnification of the boxed region of the whole pollen tube shown in the far right panel. Scale bars = 4 μm. **(B)** Corresponding single optical slices of images shown in **(A)** allowing clear visualization of single actin filament dynamics. Actin filaments are highlighted by two dots of the same color. Red arrows indicate filament elongation events, green arrows indicate filament shrinking events, and white scissors indicate severing events. Scale bar = 4 μm. **(C)** Schematic describing the dynamics of actin filaments within the apical dome. Figure adapted from Qu et al. (2013).With the permission from American Society of Plant Biologists (www.plantcell.org). For a detailed description, see the associated text and Qu et al. (2013). 1, 2, and 3 mark actin filaments that were nucleated from the membrane at the extreme apex, that moved from apex to the apical flank, and that were nucleated from the membrane at the apical flank, respectively. **(D,E)** Dynamic formation of actin bundles in the shank region. **(D)** Filament debundling events. Yellow dots highlight actin bundles that split into two bundles highlighted with red dots and green dots. The bundle marked by red dots is subjected to severing (indicted by scissors) and depolymerization. Images are a higher magnification of the boxed region shown in the far right panel. **(E)** Bundling event. Actin filaments marked by green dots and red dots were brought together via “zipping” to form the larger bundle indicated by yellow dots. Scale bars = 4 μm.

Previous studies have shown that pollen tube growth is more sensitive than cytoplasmic streaming to actin depolymerizing drugs (Gibbon et al., 1999; Vidali et al., 2001), implying that the actin structures at the tip are highly dynamic. Direct visualization of GFP-mTalin-decorated actin filaments showed that the tip-localized actin filaments, termed short actin bundles, are indeed highly dynamic, and that their dynamics are connected to the formation of actin structures in the subapical region (Fu et al., 2001; Hwang et al., 2005). Our recent visualization and quantification of individual actin filaments within the apical dome of the *Arabidopsis thaliana* pollen tubes has provided further insight into this system. Through the use of the advanced imaging technology of spinning disk confocal microscopy, we traced the dynamics of individual actin filaments and quantified their associated parameters, such as filament elongation and shortening rates, severing frequency, and other factors (Qu et al., 2013). Our observations revealed that actin filaments are constantly generated from the apical membrane of the pollen tube (**Figure 2A**; Qu et al., 2013), and that this process is most likely mediated by formins, such as AtFH5 (Cheung et al., 2010). Actin filaments originating from the extreme apex are highly dynamic. They are either turned over locally or moved to the apical flank, presumably with the membrane flow (**Figure 2B**), partially explaining why actin filaments are less abundant at the extreme apex. Our results also provide convincing evidence that exocytosis occurs at the extreme apex, supporting previous findings (Lee et al., 2008; Wang et al., 2013).

Actin filaments elongate very rapidly within the apical region (**Table 2**; Qu et al., 2013), consistent with measurements indicating that a high concentration of actin/profilin complex is present in pollen cells (Vidali and Hepler, 1997; Gibbon et al., 1999; Snowman et al., 2002). Actin filaments are also severed frequently within the apical region (**Figure 2B**; Qu et al., 2013), similar to findings showing that actin filaments in the cortical region of etiolated hypocotyl cells and BY-2 suspension cells are primarily eliminated by the filament severing activity (Staiger et al., 2009; Smertenko et al., 2010). Besides driving the local turnover of apical actin filaments, this severing activity may facilitate the departure of the severed actin filaments from the apical region, providing a pool of actin filaments that can be used for the construction of actin structures in the subapical region (**Figure 2A**; Qu et al., 2013). Thus, to some extent, actin filaments in the apical and subapical regions appear to be inherently connected, consistent with previous findings (Fu et al., 2001; Hwang et al., 2005; Cheung et al., 2010). Based on these observations, we present a simple model describing the organization and dynamics of actin filaments within the apical dome of the pollen tube (**Figure 2C**; Qu et al., 2013). A highly dynamic pool of actin filaments are constantly generated from the apical membrane. They are either turned over locally by filament severing and depolymerization activities, or they can move from the extreme apex to the apical flank, leading to decreased abundance of actin filaments in the apical region of the pollen tube (**Figure 2C**).

**Table 2:** Dynamic parameters associated with actin filaments in different regions of the pollen tube.

	Apical region	Subapical region	Shank
Maximal filament length (**μ**m)	2.5 ± 0.2^1^	3.277 ± 0.322^2^	4.63 ± 0.25^2^
Filament lifetime (s)	20.2 ± 2.9^1^	25.4 ± 1.73^2^	25.7 ± 1.2^2^
Severing frequency (breaks/**μ**m/s)	0.034 ± 0.009^1^	0.024 ± 0.005^2^	0.020 ± 0.002^2^
Elongation rate (**μ**m/s)	0.25 ± 0.02^1^	0.245 ± 0.02^2^	0.430 ± 0.021^2^
Depolymerization rate (**μ**m/s)	0.22 ± 0.01^1^	0.204 ± 0.01^2^	0.334 ± 0.022^2^
Bundling frequency (events/**μ**m^2^/s, ×10^-4^)	__	__	2.3 ± 0.54^2^
Debundling frequency (events/**μ**m^2^/s, ×10^-4^)	__	__	5.4 ± 0.65^2^

*The quantitative parameters associated with the dynamics of individual actin filaments were reported in Qu et al. (2013)^1^ and Zheng et al. (2013)^2^.*

The dynamics of actin filaments within the shank region were also traced and quantified. By comparison, maximal filament length substantially increased, and severing frequency substantially decreased in the shank region compared to that in the apical region (**Table 2**), suggesting that shank-oriented actin filaments are relatively stable. This finding is consistent with previous observations showing that cytoplasmic streaming is more resistant than pollen tube growth to actin depolymerizing drugs (Gibbon et al., 1999; Vidali et al., 2001). Given that most actin filaments are packed into bundles, presumably by “zippering” individual actin filaments together via actin bundling factors, such as villin, fimbrin, and others, actin bundling activities were analyzed in *Arabidopsis* pollen tubes (**Figures 2D,E**). Two metrics describing bundle dynamics during live-cell imaging were analyzed: the bundling and debundling frequencies (**Table 2**). Bundling was observed at a frequency of 2.3 × 10^-4^ events/μm^2^/s, whereas unbundling occurred at a frequency of 5.4 × 10^-5^ events/μm^2^/s (Zheng et al., 2013). By comparison, the bundling frequency in hypocotyl epidermal cells was measured to be 6.9 × 10^-5^ events/μm^2^/s (Hoffmann et al., 2014). These findings may explain, to some extent, why most actin filaments exist in longitudinal bundles in the shank of pollen tubes. Future documentation of the role and mechanism of action of several major actin bundling factors in these processes will shed light on the regulation of the equilibrium between individual actin filaments and bundles.

## REGULATION OF ACTIN DYNAMICS IN THE POLLEN TUBE: THE ROLES AND MECHANISMS OF ACTION OF SEVERAL POLLEN-EXPRESSED ABPs

Considering the fact that the organization and dynamics of the actin cytoskeleton are directly regulated by various ABPs, studying their functions and mechanisms of action should provide insights into the regulation of the organization and dynamics of the actin cytoskeleton in the pollen tube. Indeed, genetic manipulation of pollen-expressed ABPs has increasingly enriched our knowledge in this area. Given that actin filaments in the apical and subapical regions are highly dynamic and inherently connected (Fu et al., 2001; Hwang et al., 2005; Cheung et al., 2010; Qu et al., 2013) and that shank-oriented longitudinal actin cables are relatively stable and, to some extent, functionally distinct, in the next sections, we will review the current state of knowledge regarding several ABPs that have been implicated in regulation of actin structures either in the apical and subapical regions or in the shank region.

## SEVERAL ABPs HAVE BEEN IMPLICATED IN REGULATION OF THE CONSTRUCTION AND REMODELING OF ACTIN STRUCTURES IN THE APICAL AND SUBAPICAL REGIONS

It is now generally accepted that a population of highly dynamic actin filaments are present in the apical region of the pollen tube. However, the organizational nature of the actin structures within the apical region remains poorly understood. Recent characterization of several ABPs has provided unique insights into this question and has also substantially expanded our understanding of the functions of these actin filaments, leading us to consider how they regulate cellular processes, such as exocytosis.

### FORMIN NUCLEATES ACTIN FILAMENTS FROM THE APICAL MEMBRANE

Live-cell imaging of actin filament dynamics has shown that actin filaments are constantly generated from the apical plasma membrane (**Figure 2A**), suggesting that membrane-anchored actin nucleation factors may be required for this role. The Arp2/3 complex and formin proteins are arguably the best characterized actin nucleation factors in plants (Deeks et al., 2002; Blanchoin and Staiger, 2010; Zheng et al., 2012; Yanagisawa et al., 2013). Historically, the Arp2/3 complex was assumed to play a role in regulating the nucleation of actin filaments in the apical region of the pollen tube (Mathur and Hulskamp, 2001). However, considering the observations that actin filaments grow outward quite linearly from the apical membrane and that loss of function of the Arp2/3 complex yields no obvious phenotype in pollen (unpublished observation), it is very unlikely that the Arp2/3 complex acts as the major nucleation factor in that region. In contrast, the formins might be more reasonable candidates for actin nucleation factors in this region. Overexpression of *Arabidopsis* Formin1 (AtFH1) has been shown to result in excessive formation of actin cables and to induce membrane curvature at the pollen tube tip (Cheung and Wu, 2004), implicating this formin in the generation of actin filaments from the apical membrane. Quite recently, it was demonstrated that AtFH5 is a major regulator of the nucleation of actin filaments growing from the apical membrane of the pollen tube (Cheung et al., 2010). AtFH5 localized at the apical membrane, and knockdown of AtFH5 diminished the abundance of actin structures in the apical and subapical regions of pollen tubes (Cheung et al., 2010), suggesting that apical membrane-anchored AtFH5 nucleates actin assembly for the construction of actin structures in the apical and subapical regions. In support of this hypothesis, biochemical data revealed that AtFH5 is a bona fide actin nucleation factor and is capable of nucleating actin assembly from actin monomers or actin monomers bound to profilin (Ingouff et al., 2005). Further studies are needed to dissect how the activity of AtFH5 is regulated during pollen tube growth, as well as how it may coordinate its activity with that of other formins. Additionally, considering the fact that the formin proteins can utilize the profilin/actin complex for assembly and that the actin monomer pool in plant cells is predicted to be buffered by profilin (Staiger and Blanchoin, 2006), future analysis of the relationship between AtFH5 and profilin in the pollen tube is also necessary.

### CALCIUM-DEPENDENT FILAMENT SEVERING PROTEINS DRIVE THE TURNOVER OF APICAL ACTIN FILAMENTS

Functional characterization of villin proteins has recently provided unique insights into how the rapid turnover of actin filaments in the apical region of the pollen tube is achieved. Considering the fact that a tip-focused calcium gradient is present in the pollen tube with calcium concentrations that can reach 1–3 μM in the apical region (Pierson et al., 1994; Holdaway-Clarke et al., 1997; Messerli et al., 2000), villin, a calcium-responsive actin depolymerization promoting factor, was suggested to be an important player (Hepler et al., 2001; Vidali and Hepler, 2001; Yokota et al., 2005; Staiger et al., 2010). Villin was originally isolated from lily (*Lilium longiflorum*) pollen biochemically (Nakayasu et al., 1998; Yokota et al., 1998) and was shown to reduce the length of actin filaments in the presence of calcium/calmodulin (Yokota et al., 2005). Since then, several members of the villin/gelsolin/fragmin superfamily of proteins have been implicated in regulation of actin dynamics in the pollen tube (Xiang et al., 2007; Wang et al., 2008b). Importantly, by taking advantage of the power of *A. thaliana* genetic approaches, characterization of villins in *Arabidopsis* has provided exceptional insights into the roles of these proteins in regulating actin dynamics in the pollen tube and during its growth (Huang et al., 2014).

We have previously shown that pollen-expressed *Arabidopsis* villin2 (VLN2) and VLN5 are able to sever actin filaments in the presence of micromolar concentrations of free calcium (Zhang et al., 2010a; Bao et al., 2012). Remarkably, we also found that VLN5 promotes actin depolymerization in the presence of profilin under similar conditions (Zhang et al., 2010a), leading to the hypothesis that villins may regulate actin dynamics by promoting actin depolymerization in the apical region. Consistent with this idea, actin filaments accumulated in the apical region of *vln2 vln5* double mutant pollen tubes (Qu et al., 2013). Visualization of the dynamics of actin filaments at a single filament resolution showed that the average filament severing frequency decreased and the average maximum filament lifetime increased in the apical region of *vln2 vln5* double mutant pollen tubes (Qu et al., 2013). These results suggest that villins promote actin turnover via their calcium-dependent filament severing activity at pollen tube tips.

Consistent with the idea that actin filaments nucleated from the apical membrane are required for the construction of actin structures at the subapical region (Cheung et al., 2010), the accumulation of actin filaments in the apical region of *vln2 vln5* double mutant pollen tubes was accompanied by disorganization of actin filaments at the subapical region (Qu et al., 2013). Certainly, as villins are known to be versatile actin regulatory proteins (Huang et al., 2014), the filament bundling and stabilizing activity of VLN2 and VLN5 may also contribute to the construction of actin structures in the subapical region of the pollen tube. In support of this idea, actin filaments in the subapical region of *vln2 vln5* double mutant pollen tubes are more wavy and are thinner than those in their wild-type counterparts (Qu et al., 2013). Additionally, villin-mediated filament severing activity has also been implicated in regulation of the construction of the subapical region by eliminating actin filaments that do not align longitudinally (Qu et al., 2013).

Several recently characterized calcium-responsive filament severing proteins, such as MAP18 (Zhu et al., 2013) and MDP25 (Qin et al., 2014), may coordinate with villins to regulate actin dynamics in the pollen tube. Both MAP18 and MDP25 were shown to function as microtubule-associated proteins in vegetative cells (Wang et al., 2007; Li et al., 2011). Surprisingly, these proteins have been shown to act as regulators of actin dynamics in the pollen tube (Zhu et al., 2013; Qin et al., 2014). *In vitro* biochemical characterization revealed that both proteins are able to sever actin filaments in a calcium-dependent manner (Zhu et al., 2013; Qin et al., 2014). Direct visualization of the actin cytoskeleton showed that more actin bundles were present in the apical and subapical region of *map18* pollen tubes compared to the wild-type (Zhu et al., 2013), suggesting that MAP18 drives the turnover of actin filaments by severing. Similarly, filament severing frequency was decreased, and actin filaments were more abundant in the subapical region of *mdp25* pollen tubes (Qin et al., 2014). How these calcium-responsive filament severing proteins coordinate to regulate the turnover of actin filaments at the pollen tube apex remains to be determined. Additionally, actin-depolymerizing factor (ADF) and actin-interacting protein (AIP1) have been implicated in the regulation of actin structures at the subapical region of the pollen tube (Chen et al., 2002; Lovy-Wheeler et al., 2006), although how they coordinate with calcium-responsive filament severing proteins to drive the turnover of actin filaments remains to be addressed.

## SEVERAL ABPs HAVE BEEN IMPLICATED IN THE GENERATION AND REMODELING OF LONGITUDINAL ACTIN CABLES IN THE SHANK

In the shank region, actin filaments are arranged into longitudinal cables (Lancelle and Hepler, 1992; Gibbon et al., 1999; Fu et al., 2001; Lovy-Wheeler et al., 2005). It is clear that these longitudinal actin cables regulate cytoplasmic streaming in pollen tubes by providing molecular tracks for myosins, similar to observations in other plant cells (Shimmen and Yokota, 2004). Previous results have shown that cytoplasmic streaming is relatively more resistant than pollen tube growth to actin depolymerizing drugs (Gibbon et al., 1999; Vidali et al., 2001), suggesting that longitudinal actin cables are relatively stable. However, the mechanisms by which these longitudinal actin cables are generated and maintained, as well as how they are remodeled, remain largely unknown. Characterization of several pollen-expressed ABPs, including several recently characterized in *Arabidopsis*, has shed new light on these mechanisms.

### FORMINS NUCLEATE ACTIN ASSEMBLY FOR THE CONSTRUCTION OF LONGITUDINAL ACTIN CABLES

Characterization of the pollen-specific Class I formin, AtFH3, has provided insight into the nucleation step required for the generation of longitudinal actin cables in the shank of the pollen tube (Ye et al., 2009). AtFH3 is a bona fide actin nucleation factor, capable of using the actin/profilin complex to nucleate actin assembly. It is also able to interact with the barbed end of actin filaments. Knockdown of *AtFH3* using RNAi affects the formation of longitudinal actin cables, resulting in depolarized growth of the pollen tube (Ye et al., 2009). Given that AtFH3 is an important regulator of actin nucleation, it will be very interesting to better understand exactly how its activity is regulated. Furthermore, characterization of how AtFH3 coordinates with other formins to nucleate actin assembly in the shank region should be a subject of future investigation.

### ADF/COFILIN REGULATES THE REMODELING OF LONGITUDINAL ACTIN CABLES

Actin-depolymerizing factor/cofilin is important for driving the rapid turnover of actin filaments in cells. Members of the ADF family have been implicated in regulation of actin turnover in pollen. For example, lily ADFs were shown to accumulate at the germination aperture during tube protrusion, but distribute evenly in the pollen tube (Smertenko et al., 2001). In contrast to this observation, tagging of tobacco pollen-specific ADF/cofilin (NtADF1) with GFP revealed that this protein decorates filamentous actin, particularly subapical actin structures and longitudinal actin cables in the shank (Chen et al., 2002). Overexpression of NtADF1 reduces the number of axially arranged fine actin cables (Chen et al., 2002), implicating ADF as a driver in the turnover of longitudinal actin cables.

In order to probe the intracellular localization of pollen-specific *Arabidopsis* actin-depolymerizing factor 7 (ADF7) in the pollen tube, we generated several ADF7-EGFP fusion constructs containing EGFP inserted in different locations within the ADF7 molecule in hopes of minimizing the interference of the EGFP fusion on the function of ADF7. Among which, one ADF7-EGFP fusion protein (ADF7-EGFP_V 10_) is fully functional (Zheng et al., 2013) and decorates filamentous actin throughout the pollen tube (Daher et al., 2011; Zheng et al., 2013). *In vitro* biochemical characterization showed that ADF7 is a typical ADF; it prefers ADP-loaded actin and inhibits nucleotide exchange, and it is able to promote actin depolymerization and sever actin filaments. However, by comparison, its actin depolymerizing and severing activity are lower than that of the vegetative ADF1 (Zheng et al., 2013). This observation is consistent with a previous report that the lily pollen ADF1 (LIADF1) has weak actin depolymerizing activity compared to the vegetative *Zea maize* ADF, ZmADF3 (Smertenko et al., 2001). These data suggest that reproductive ADFs may have evolved to play specific roles in the regulation of actin dynamics in the context of pollen.

Interestingly, ADF7-EGFP_V 10_ was found to fully retain both the monomer actin (G-actin) binding and filament severing activities and are fully functional *in vivo*, whereas another ADF7-EGFP fusion protein (ADF7-EGFP_D75_) retained G-actin binding, but was deficient in severing actin filaments, was non-functional in the pollen tube (Zheng et al., 2013). Results from analysis of these proteins suggested that the severing activity of ADF7 is crucial for its functions *in vivo*. Consistent with this hypothesis, specific abolishment of the severing activity of yeast cofilin has been shown to affect its *in vivo* function (Chen and Pollard, 2011). These data suggest that the associated severing activity is important for the function of ADF/cofilin protein family members *in vivo*. Additional observations showed that the turnover rate of actin filaments was decreased in *adf7* pollen tubes, consistent with a role in promotion of actin depolymerization. Consequently, the amount of filamentous actin and the extent of filament bundling were increased in *adf7* pollen tubes. These data suggest that ADF7 is an important player in driving the turnover of actin filaments in the shanks of pollen tubes (Zheng et al., 2013). Detailed documentation of the functional coordination of ADF7 with other ADF isovariants, such as ADF10 (Daher et al., 2011), will provide further insight into the regulation of actin turnover in the pollen tube. Additionally, determination of the functional relationship between ADF7 and other players, such as cyclase-associated protein (CAP1) (Chaudhry et al., 2007; Deeks et al., 2007) and AIP1 (Ketelaar et al., 2004a; Shi et al., 2013) will also shed light on the regulation of the dynamic turnover of longitudinal actin cables.

### ACTIN FILAMENT BUNDLING PROTEINS GENERATE LONGITUDINAL CABLES AND MAINTAIN THEIR LONGITUDINAL ARRANGEMENT

Several actin filament bundling proteins have been implicated in organization of actin filaments into bundles, as well as maintenance of the longitudinal arrangement of actin bundles in the shanks of pollen tubes. *In vitro* biochemical studies have shown that *Arabidopsis* FIMBRIN5 (FIM5) is a bona fide actin bundling factor that stabilizes actin filaments (Wu et al., 2010). Loss of function of FIM5 affects pollen germination and polarized tube growth. FIM5 decorates actin filaments throughout the pollen tube, and loss of function of FIM5 results in disorganization of actin filaments in the pollen tube and alters the longitudinal arrangement of actin cables (Wu et al., 2010; Su et al., 2012). As a result, the pattern of cytoplasmic streaming is altered, exhibiting decreased velocity and altered direction (Wu et al., 2010). Unexpectedly, actin bundles were found to be thicker in *fim5* pollen tubes compared to wild-type tubes (Wu et al., 2010), suggesting that loss of function of FIM5 may upregulate the activity some other actin bundling factors. This question might be worthwhile to examine in the future. Furthermore, the activity of LI-FIM1 was shown to be sensitive to pH (Su et al., 2012), suggesting that fimbrin might act as a sensor that regulates actin dynamics in response to pH. Further study is needed to characterize the mechanisms by which fimbrin regulates actin dynamics in the pollen tube in response to oscillations in intracellular pH.

The bundling factor villin has also been implicated in regulation of longitudinal actin bundle formation in the shank of the pollen tube. Two pollen-expressed *Arabidopsis* villin isovariants, VLN2 and VLN5, were demonstrated to be bona fide actin filament bundling proteins (Zhang et al., 2010a; Bao et al., 2012). Though loss of function of VLN5 alone did not have an overt effect on the generation and formation of longitudinal actin cables (Zhang et al., 2010a), loss of function of both VLN2 and VLN5 decreased the amount of actin filaments, suggesting that these proteins stabilize actin filaments in the pollen tube. Additionally, actin cables became thinner and more disorganized in the shanks of *vln2 vln5* pollen tubes (Qu et al., 2013), suggesting that VLN2 and VLN5 function as actin bundling factors that regulate the formation of shank-oriented longitudinal actin bundles.

Several other actin filament bundling factors may also be involved in regulating the construction and maintenance of longitudinal actin cables, such as LIMs (Papuga et al., 2010) and the recently identified, novel, plant actin-crosslinking protein, CROLIN1 (Jia et al., 2013). For instance, LI-LIM1 was shown to promote actin filament bundling and stabilize actin filaments in the pollen tube (Wang et al., 2008a), suggesting that LI-LIM1 is involved in regulating the formation of longitudinal actin cables. Loss of function of CROLIN1 led to instability of actin filaments in the shanks of pollen tubes (Jia et al., 2013), implicating this protein in regulation of the construction of shank-oriented, longitudinal actin cables. However, the means by which CROLIN1 regulates the construction and dynamics of longitudinal actin cables needs to be carefully examined.

### SCHEMATIC DESCRIBING THE REGULATION OF THE CONSTRUCTION AND REMODELING OF DISTINCT ACTIN STRUCTURES IN THE POLLEN TUBE

**FIGURE 3:**
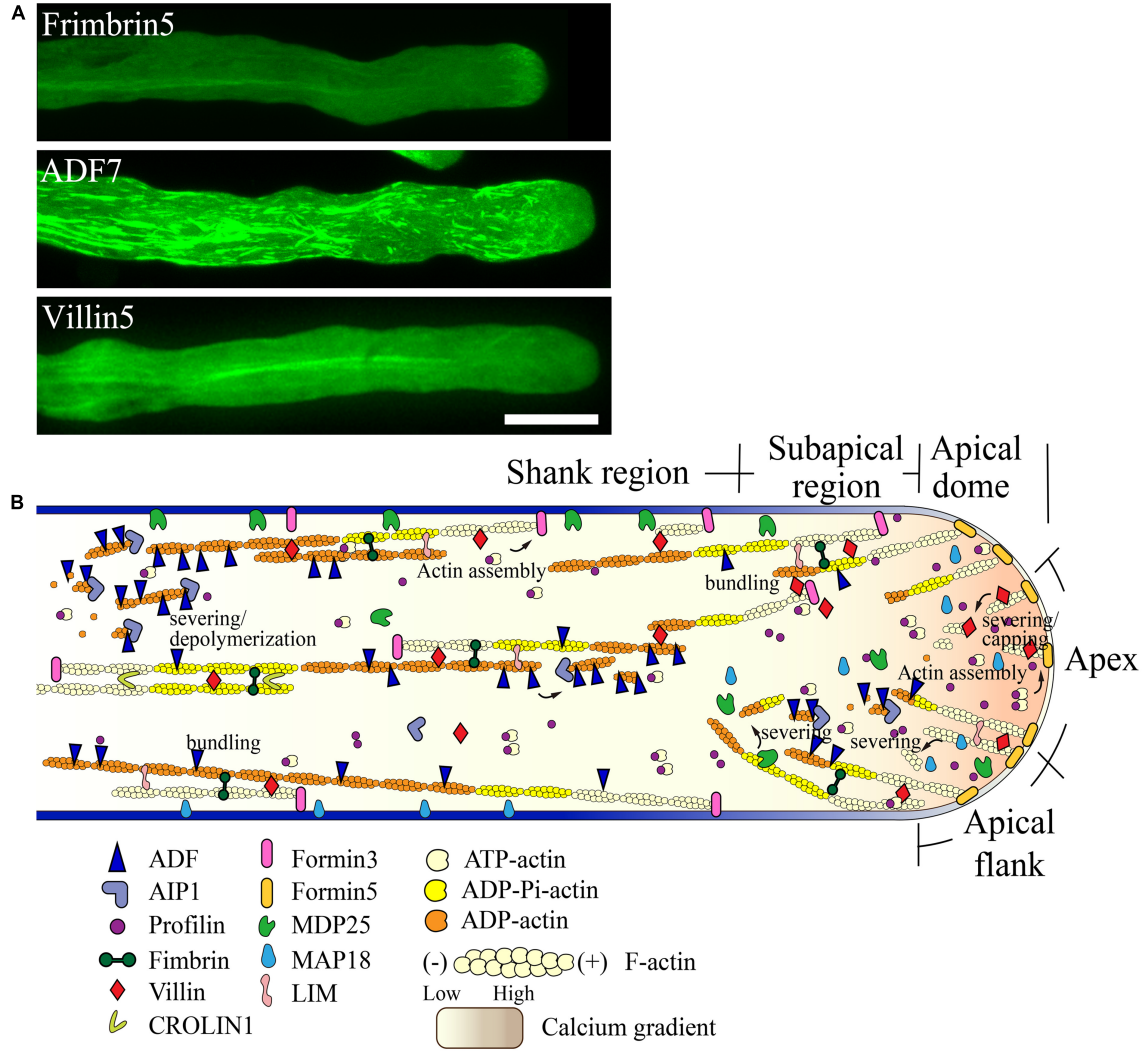
**Schematic describing the regulation of actin dynamics in the pollen tube based on the functional characterization of ABPs derived mainly from *Arabidopsis.* (A)** Intracellular localization of several ABPs in the pollen tube. For methods used to determine the localization of FIMBRIN5, ADF7, and VILLIN5, see descriptions in previous studies ( Wu et al., 2010; Qu et al., 2013; Zheng et al., 2013). Scale bar = 10 μm. **(B)** Schematic describing the intracellular localization and function of various ABPs in the pollen tube. For detailed information regarding the intracellular localization and function of each ABP, see the description in the text.

As described above, our knowledge regarding the organization and regulation of the actin cytoskeleton in the pollen tube has grown substantially. Functional characterization of several pollen-expressed ABPs has enriched our understanding of the relevant mechanisms. In particular, direct visualization and quantitative analysis of the dynamics of individual actin filaments in pollen tubes with loss of function of specific ABPs, as well as careful comparisons with wild-type pollen tubes have yielded substantial insight. Based on these data, we propose a simple model describing the role of various ABPs in regulating the organization of the actin cytoskeleton in the pollen tube (**Figure 3**).

Two formins, AtFH5 and AtFH3, regulate the construction of actin structures in the apical and subapical regions and shank region, respectively. AtFH5 is localized on the apical membrane within the apical dome, where it nucleates actin filaments from the apical membrane that are used for the construction of actin structures in the apical and subapical regions (**Figure 3B**; Cheung et al., 2010). In contrast, AtFH3 nucleates actin filaments within the cytoplasm or from the membrane to generate longitudinal actin cables in the shank (**Figure 3B**; Ye et al., 2009). Certainly, transport of actin filaments from the apical and subapical regions to the shank could represent another potential mechanism leading to the construction of longitudinal actin cables. Additionally, other pollen-expressed formin proteins may play yet undiscovered roles in construction of distinct actin arrays in the pollen tube.

To build actin structures in the apical and subapical regions, AtFH5-generated actin filaments are instantaneously bundled by actin bundling factors, such as villins (Qu et al., 2013), fimbrins (Wu et al., 2010; Su et al., 2012), and/or LIMs (Papuga et al., 2010), allowing them to grow outward linearly from the membrane. These actin filaments are subjected to rapid turnover due to filament severing and depolymerizing activities. With respect to these activities, several calcium-dependent filament severing and depolymerizing proteins, including villins (Qu et al., 2013), MAP18 (Zhu et al., 2013), and MDP25 (Qin et al., 2014), are reasonable candidates for these roles. Together, these mechanisms presumably lead to the decreased abundance of actin filaments at the pollen tube apex. Certainly, a potential role for ADF and its cofactors in promoting the turnover of actin filaments in the apical region needs to be examined in the future.

Filament severing activity mediated by calcium-responsive severing proteins may allow the departure of actin filaments originating from the apical membrane away from the apical region. These filaments can then be used for the construction of subapical actin structures. This idea is partially supported by the observation that loss of function of villins affects the formation of actin structures at the subapex (Qu et al., 2013). Furthermore, actin filaments originating from the extreme apical membrane can shift toward the apical flank via membrane flow and can further elongate to directly participate in the construction of actin structures at the subapex. At the subapical region, villin plays a major role in the formation of regular actin collars through bundling and stabilization of longitudinally-aligned actin filaments (**Figure 3B**; Qu et al., 2013). Additionally, villin may facilitate the formation of regular actin collars by eliminating actin filaments that do not align longitudinally via its filament severing activity (**Figure 3B**; Qu et al., 2013). MDP25 may also have a similar function in severing of actin filaments that do not align longitudinally in the subapical region (Qin et al., 2014). Furthermore, previous studies suggest that ADF and AIP1 may also be involved in regulating the turnover of actin structures at the subapical region (Chen et al., 2002; Lovy-Wheeler et al., 2006).

In the shank, AtFH3-generated actin filaments (Ye et al., 2009) initially undergo dynamic turnover due to the activity of ADFs, such as ADF7 (Zheng et al., 2013), along with several ADF cofactors, like AIP1 (Ketelaar et al., 2004a; Shi et al., 2013) and CAP1 (Chaudhry et al., 2007; Deeks et al., 2007). Subsequently, actin bundling factors, such as fimbrins, villins, LIMs, and CROLIN1, may participate in packing these filaments into longitudinal actin cables in addition to maintaining their longitudinal arrangement and stabilizing them (**Figure 3B**).

## CONCLUDING REMARKS

There is no doubt that our knowledge regarding the organization, dynamics, and regulation of the actin cytoskeleton in the pollen tube has grown substantially in recent years, although many issues remain to be resolved. Importantly, adoption of the *A. thaliana* pollen tube as a cellular system for the study of these processes has greatly facilitated progress in this field due to the powerful combination of *Arabidopsis* genomic and genetic approaches, as well as the introduction of complex spatiotemporal imaging technology and the development of appropriate actin markers that have allowed real-time visualization of individual actin filaments. Recent improvements in imaging technology have expanded our view of actin filament dynamics, as well as our understanding of the underlying organization of actin structures in the pollen tube. A detailed description of the dynamic properties of actin filaments in the apical region represents one of the exciting achievements resulting from these technologies (Qu et al., 2013), and this data has greatly enhanced our understanding of the function of the actin cytoskeleton in regulating polarized pollen tube growth. Careful characterization of the mode of action of several pollen-expressed ABPs has provided additional insights into regulation of the actin cytoskeleton during pollen tube growth. As some ABPs act as direct sensors for various signals, these studies have also shed light on how various signals converge on these ABPs to control actin dynamics. For example, Ca^2+^ signaling has been shown to regulate actin dynamics by controlling the activity of villins in the pollen tube. An important future challenge will be to delineate the roles of various signaling transduction pathways in order to determine how various signals converge on ABPs to regulate actin dynamics in the pollen tube. Certainly, in this field, the eternal and most challenging question is still to understand precisely how the actin cytoskeleton functions to regulate the pollen tube growth.

## Conflict of Interest Statement

The authors declare that the research was conducted in the absence of any commercial or financial relationships that could be construed as a potential conflict of interest.
